# Effect of Homogenization Treatment on Microstructure, Dendritic Segregation, and Primary Carbides in H13 Steel

**DOI:** 10.3390/ma18204785

**Published:** 2025-10-20

**Authors:** Xijie Wang, Huan Yu, Sibo He

**Affiliations:** Xingfa School of Mining Engineering, Wuhan Institute of Technology, Wuhan 430205, China; 22417010027@stu.wit.edu.cn (H.Y.); 22417010050@stu.wit.edu.cn (S.H.)

**Keywords:** H13 steel, homogenization, dendritic segregation, primary carbides, grain size

## Abstract

In the current work, the dendritic structure evolution, primary carbide dissolution, and austenite grain growth during the homogenization process of H13 steel at 1150–1250 °C were investigated to achieve a balanced improvement in segregation, primary carbide, and grain size controlling. The homogenization kinetic model was established to predict the soaking time needed for eliminating the dendritic structures. The results show that dendritic structures disappear gradually during the homogenization process. The time required for eliminating the dendritic structures of the investigated H13 steel at 1250 °C is 600 min, and those at 1150 and 1200 °C are estimated to be 3.31 and 1.78 times longer than that at 1250 °C. The Mo-rich primary carbides in H13 steel decompose and dissolve completely at the investigated temperature range. However, the V-rich primary carbides could not dissolve completely even at 1250 °C. The decreases in Ti and N content in steel are beneficial for lowering the complete dissolution temperature of V-rich primary carbides. The austenite grains grow slowly at 1150 °C, and become abnormally coarser at 1200–1250 °C.

## 1. Introduction

H13 (4Cr5MoSiV1) steel is widely applied in die casting molds, hot forging molds, and aluminum extrusion molds for its good strength, hardness, toughness, and thermal stability [1,2,3]. It usually works at high temperatures, high pressures, and cyclic loading conditions, and is sensitive to thermal fatigue [1,4]. During the solidification process, the alloying elements with the partition coefficient less than one (like the main carbide-formation alloying elements C, Cr, Mo, and V in H13 steel) will be enriched in the liquid phase, which causes the dendritic segregation. Moreover, the enriched alloying elements provide the driving force for the generation of primary carbides in the residual liquid phase within the inter-dendrite region of H13 steel [5,6,7,8]. In addition, the enriched alloying elements cause more pronounced secondary carbides to precipitate in the inter-dendrite region. The inhomogeneous secondary carbides in the as-cast steel will exist in the form of the banded structures after the hot forming process [9,10,11]. The primary carbides and banded structures will cause the stress concentration during the service of H13 steel, and promote the initiation and propagation of thermal fatigue cracks [11,12,13,14]. Thus, eliminating the dendritic segregation in H13 steel is essential for prolonging the service life of hot working dies.

Previous studies paid more attention to the solidification condition improvement [15,16,17] and modifier addition [10,18,19] to suppress the dendritic segregation. However, the methods mentioned above can mitigate the compositional inhomogeneity and large carbides caused by dendritic segregation, but they cannot eliminate these defects completely. High-temperature homogenization treatment can be applied to as-cast steel to eliminate the large-sized primary carbides and inhomogeneity caused by dendritic segregation. In the previous studies, high-temperature homogenization treatment of H13 steel is typically performed at 1100–1300 °C. Furthermore, the soaking time is adjusted based on the degree of segregation in the steel. Ma et al. [11] reported that the dendritic segregation in the forged H13 steel were eliminated after homogenization at 1200 °C for 8 h. The transverse impact toughness of the homogenized H13 steel was improved compared to the untreated one. Han et al. [20] found that the H13 steel experienced overheat when the homogenization temperature exceeded 1230 °C, and the microhardness of steel after homogenization decreased with the increasing temperature. Huang et al. [21] reported that V-rich primary carbides started to dissolve when the homogenization temperature exceeded 1150 °C, and Ti improved their thermal stability. The segregation ratio of Cr, Mo, and V elements in the H13 steel, derived as the maximum-to-minimum elemental concentration ratio across the secondary dendrite arms, did not drop to unity even after soaking for over 30 h at 1200–1300 °C [22]. This implied that the complete homogenization of the segregated elements is energy-intensive and hard to be realized. Thus, it was proposed that the terminus of homogenization treatment is supposed to be the time that the dendrite structures disappear [6].

The high-temperature homogenization treatment is energy-intensive and makes the treated materials prone to the abnormal growth of grains. Therefore, a proper homogenization process should be based on the actual requirements, and comprehensively consider the grain growth, dissolution of primary carbides, and dendritic segregation. In our present work, the evolution of dendrite structures, and the segregation elements changes during homogenization at 1150 to 1250 °C, were investigated via electronic probe micro-analyzer (EPMA) to establish the homogenization kinetic model for estimating the needed soaking time for eliminating the dendrite structures. The morphology and area fraction of primary carbides were studied to observe the dissolution behavior. The size of austenite grains varying with soaking temperature and time were measured to establish the growth kinetics model for predicting the grain growth during homogenization treatment. The present work aims to provide a theoretical basis for the high-temperature homogenization of H13 steel in production.

## 2. Materials and Methods

This study investigated an as-cast H13 ingot processed using a 1-ton electroslag remelting (ESR) furnace. The chemical compositions of the investigated H13 steel are as follows (mass%): 0.41 C, 0.86 Si, 0.36 Mn, 1.05 V, 1.32 Mo, 5.27 Cr, 0.018 Al, 0.0059 Nb, 0.0035 Ti, 0.0038 O, 0.0040 S, and 0.020 N. To ensure sampling locations remain as consistent as possible, a 20 mm thick disk was cross-sectioned from a position 10 cm below the top of the 200 mm diameter electroslag ingot. Subsequently, specimens with a size of 10 mm × 10 mm × 4 mm were taken from the mid-radius (1/2 radius) location of the disk. The samples were then heated in a resistance furnace in the protective Ar gas atmosphere at 1150, 1200, and 1250 °C, and held for 10 to 720 min. Approximately 10 L of distilled water in an iron barrel under the ambient temperature around 25–30 °C was used for quenching high-temperature specimens. The specimens were taken out of the furnace after finishing the homogenization, thrown into the water immediately, and picked up from the water after about 60 s.

After being ground and polished, the specimens were etched by 4% nital at room temperature for about 30–60 s to reveal the dendritic structures. The dendritic structures were observed via an optical microscope (OM, DSX 510, Olympus Co., Tokyo, Japan) at dark-field mode. After that, the specimens were polished again and examined by EMPA (EPMA-8050G, Shimadzu, Japan) with accelerate voltage of 15.0 kV, beam current of 0.1 μm, and scanning rate of 20 ms/point to investigate the elements distribution of the specimens. Subsequently, fifty images of each specimen from random fields of view at 1000× magnification were taken by scanning electron microscope (SEM, Nova 400 Nano, FEI Co., Hillsboro, OR, USA), to perform a statistical analysis on the two-dimensional areas and average size of the primary carbides. The three-dimensional (3D) morphology of primary carbides was obtained through the non-aqueous electrolytic method, and observed by SEM. The morphologies and compositions of the primary carbides were determined by SEM equipped with an energy-dispersive spectrometer (EDS, Le350 Penta FETx-3, Oxford Instruments Co., Abingdon, UK). To reveal the austenite grains, the samples were polished again. Next, the specimens were etched with saturated picric acid added with sodium dodecylbenzene sulfonate at 80 °C for 30 to 60 s, and then gently wiped on the polishing cloth under running water. The etching and wiping step were repeated three to five times until the grain boundaries can be seen clearly. Afterwards, the specimens were photographed by the OM. The grain sizes were automatically measured by the Image-Pro Plus software 6.0, and the average grain size was the average equal-area-circle diameter.

## 3. Results and Discussion

### 3.1. Evolution of Dendritic Structures During the Homogenization Process

**Figure 1:**
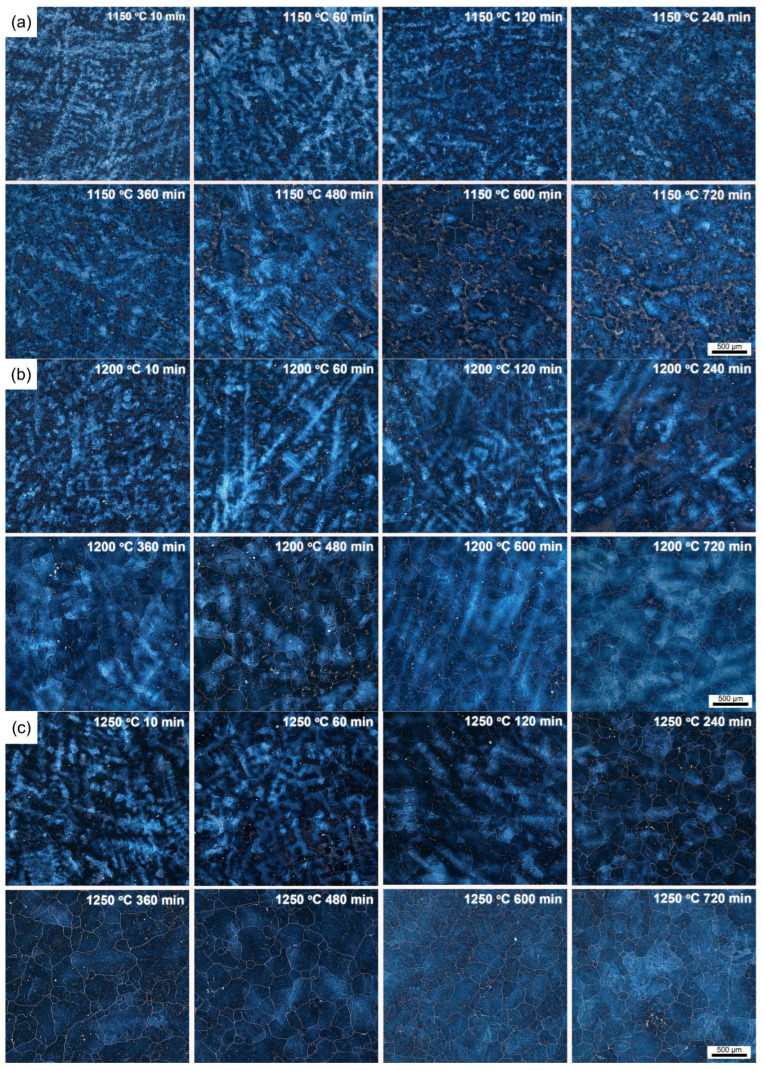
Evolution of dendritic structures during the homogenization process: (**a**) 1150 °C, (**b**) 1200 °C, and (**c**) 1250 °C.

The dendritic structures evolution of the investigated H13 steel during the homogenization process was observed in the dark-field mode of OM, as shown in Figure 1. The dendritic structure is a result of selective crystallization during the solidification process. The inter-dendritic regions typically contain a higher concentration of alloying elements and consequently more precipitates. After etching, the inter-dendritic regions and dendrite arms exhibit distinct visual characteristics under an optical microscope. In Figure 1, the dark areas represent the inter-dendritic regions, while the bright areas correspond to the dendrite arms. As the holding time increases, the segregated elements gradually diffuse, causing the boundaries between the dendrite arms and inter-dendritic regions to become progressively less distinct under OM. The dendritic structures of H13 could be clearly visualized upon soaking at 1150 °C for 10 min, and become gradually blurred with the soaking time due to the diffusion of enriched alloying elements. Meanwhile, the dendritic structures are still present even after soaking at 1150 °C for 720 min, which may be due to the limited diffusion effect at this temperature. When the soaking temperature increased to 1200 °C, the dendritic structures vanished faster because of the enhanced diffusion effect. However, it still exists after being soaked at 1200 °C for 720 min. With the temperature increased to 1250 °C, the dendritic structures disappear completely after being soaked for 600 min.

The distribution of solutes along the inter-dendritic regions can be approached by Fourier series components according to Shewman [23] as follows:

(1) $$C(x)=\bar{C}+A_{0}\cos\frac{2\pi x}{L}$$

where $\bar{C}$ is the average concentration of the alloying elements, *x* stands for the distance between the element and the grain boundary, and *L* is the secondary dendritic arm spacing, while *A*_0_ is the initial amplitude of the elemental segregation, which can be expressed as follows:

(2) $$A_{0}=\frac{1}{2}\left(C_{\mathrm{Max}}-C_{\mathrm{Min}}\right)=\frac{1}{2}∆C_{0}$$

where *C*_Max_ and *C*_Min_ are the maximum and minimum initial concentrations of the elements along the inter-dendritic spaces. The purpose of the homogenization process is to eliminate the composition difference to a desired degree. The amplitude, namely the composition difference between the dendritic arm and inter-dendritic region, decreases with increasing hold time during the homogenization. According to the second Fick’s law, *A*(*t*) is a function of holding time as follows:

(3) $$∆C_{0}\left(t\right)=A_{0}\exp\left(\frac{-4\pi^{2}}{L^{2}}Dt\right)$$

where *D* is the diffusion coefficient of the element in materials. The diffusion coefficient can be expressed as:

(4) $$D=D_{0}\exp\left(-\frac{Q}{RT}\right)$$

where *Q* is the diffusion activation energy (J·mol^−1^), R is the gas constant (8.314 J·mol^−1^·K^−1^), and *T* is the soaking temperature (K). Because of the high concentration of Cr in H13 steel, the homogenization process is believed to be limited by the diffusion of Cr [6,24]. The austenitizing process of H13 steel occurs between 860 and 915 °C [25]. Thus, the diffusion activation energy *Q* and the diffusion coefficient *D*_0_ of Cr in γ-Fe are 218,991 J·mol^−1^ and 0.0012 cm^2^·s^−1^ [26], respectively.

By combining Equations (3) and (4), *A*(*t*) can be further expressed as follows:

(5) $$A\left(t\right)=A_{0}\exp\left[\frac{-4D_{0}\pi^{2}t}{L^{2}}\exp\left(-\frac{Q}{RT}\right)\right]$$

In the relevant literature [6,24,27], it is assumed that one element is homogeneous when the amplitude decreases to 1% of the *A*_0_. Namely, the homogenization is completed as *A*(*t*)/*A_0_* = 0.01. Therefore, the soaking time for homogenization can be estimated under this assumption as follows:

(6) $$t=\frac{4.6L^{2}}{4\pi^{2}D_{0}\exp\left(-\frac{Q}{RT}\right)}$$

**Figure 2:**
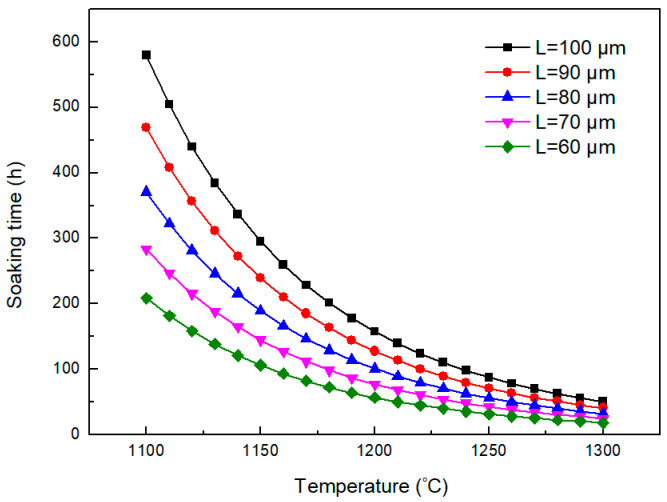
Influence of the soaking temperature and secondary dendritic arm spacing on the soaking time for complete homogenization.

According to Equation (6), the relationship between complete homogenization temperature and soaking time for alloying elements Cr is shown in Figure 2. It indicates that the soaking time decreases with the increase in homogenization temperature for the enhanced diffusion rate. However, the soaking time increases with the increase in secondary dendritic arm spacing because of the longer diffusion distance. The secondary dendritic arm spacing decides the diffusion path for the diffusion of enriched alloying elements in inter-dendritic regions to the dendritic arm regions. Thus, the larger the secondary dendritic arm spacing is, the more difficult the diffusion is. The secondary dendritic arm spacing of the investigated position in the H13 ingot is measured as 104 μm. According to the estimated results in Figure 2, the soaking time for complete homogenization of Cr is in the range of 295~87 h when the homogenization temperature ranges from 1150 °C to 1250 °C. Thus, complete homogenization is too energy-intensive to be feasible. A more feasible outcome may be the elimination of dendritic structures, rather than of elemental segregation.

**Figure 3:**
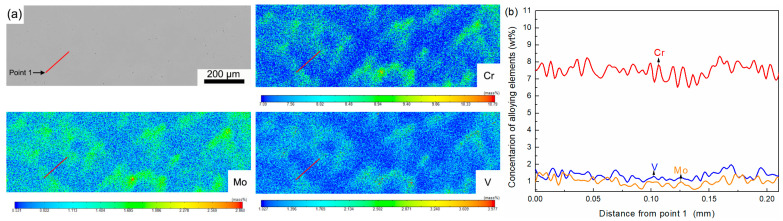
Distribution of alloying elements in H13 steel detected by EPMA: (**a**) Element mapping of steel matrix, and (**b**) line scanning of secondary dendrite arm.

**Figure 4:**
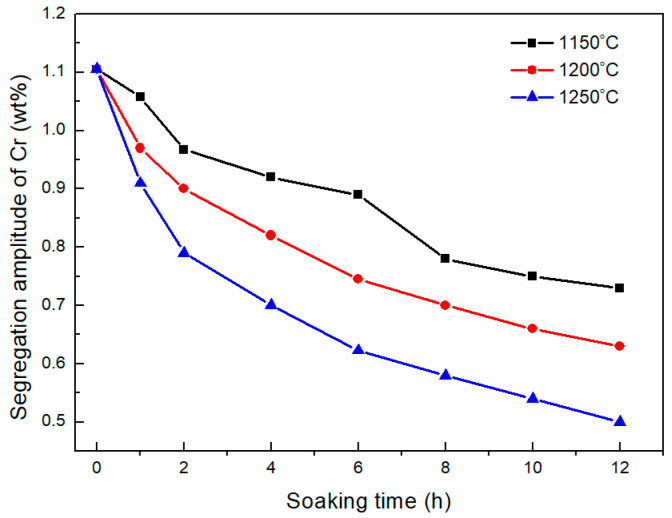
Variation in segregation amplitudes of Cr with soaking time in temperature range of 1150 to 1250 °C.

To obtain the values of elemental segregation amplitudes in the investigated H13 during the homogenization process, the distribution of alloying elements in the matrix of the studied H13 steel determined by EPMA is shown in Figure 3. To represent the distribution of alloying elements along the inter-dendritic and dendritic regions, the starting point of EPMA line scanning is marked as point 1 in Figure 3a. The defects in H13 steel resulting from dendritic segregation are coarse carbides and an inhomogeneous carbide distribution. Cr, Mo, and V are strong carbide-forming elements. In the investigated H13 steel, their total content is close to 8 mass%, and the primary goal of the homogenization treatment is to eliminate their segregation. The distributions of Cr, Mo, and V along the inter-dendritic and dendritic regions are shown as Figure 3b. The inter-dendritic regions have higher Cr, Mo, and V contents than the dendritic arms and trunks. In addition, Cr has higher segregation amplitudes than that of Mo and V. The elemental segregation amplitudes of Cr varying with homogenization temperature and soaking time are shown in Figure 4. The elemental segregation amplitudes decrease with increased soaking time. Higher homogenization temperature is beneficial for accelerating the decrease in segregation amplitudes. The dendritic structures are eliminated when soaked at 1250 °C for 600 min as shown in Figure 1. The elemental segregation amplitudes of Cr when soaked at 1250 °C for 600 min (*A_E_*) are 0.54 wt%, respectively. The values of *A_E_*/*A*_0_ of Cr are 0.48. By substituting *A_E_*/*A*_0_ into Equation (5), Equation (7) can be obtained as follows:

(7) $$t=\frac{0.73L^{2}}{4\pi^{2}D_{0}\exp\left(-\frac{Q}{RT}\right)}$$

**Figure 5:**
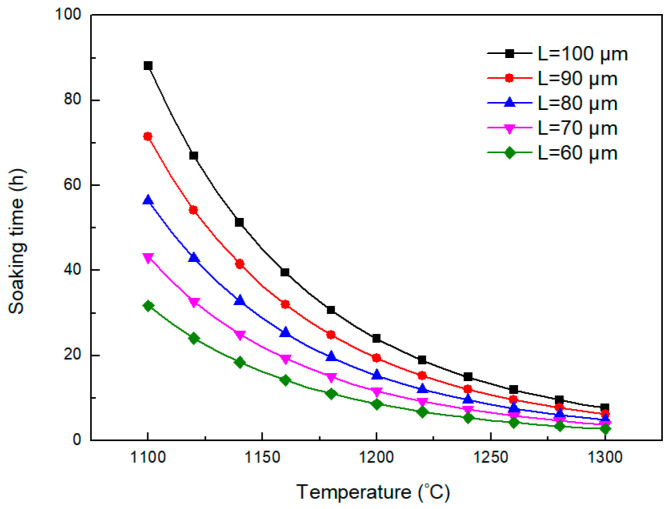
Required soaking time for elimination of dendritic structures varying with soaking temperature and secondary dendritic arm spacing.

The relationship between secondary dendritic arm spacing, homogenization temperature, and soaking time for eliminating dendritic structures can be estimated by Equation (7), as shown in Figure 5. The soaking time for eliminating the dendritic structures of the investigated H13 steel at 1250 °C is estimated as 714 min, which is close to the experimental value. It indicates that this model could be used to predict the soaking time needed for eliminating the dendritic structures. Based on this model, the soaking times at 1200 °C and 1150 °C are estimated to be longer than that at 1250 °C for 1.78 and 3.31 times. Higher temperatures provide better thermodynamic and kinetic conditions for the diffusion of Cr. The increasing soaking temperature can significantly shorten the holding time. In addition, decreasing the secondary dendritic arm spacing can effectively reduce the homogenization soaking time due to the decrease in diffusion distance. Therefore, the improvement in the solidification quality also helps to shorten the holding time.

### 3.2. Dissolution Behavior of Primary Carbides

**Figure 6:**
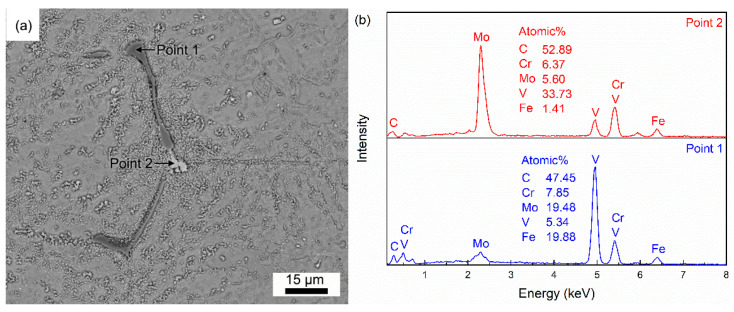
V-rich and Mo-rich primary carbides in H13 steel: (**a**) SEM images and (**b**) EDS analysis.

There are large-sized V-rich and Mo-rich primary carbides in H13 steel, which are identified as MC type and M_2_C type carbides, respectively [8]. Figure 6 shows the V-rich and Mo-rich primary carbides in the as-cast H13 steel. The V-rich primary carbides have a black or gray color under the SEM observation in the back-scatter mode, due to their lower atomic number compared to the matrix. The type of primary carbide can be identified by the difference in the color between V-rich and Mo-rich primary carbides under SEM observation in the back-scatter mode.

**Figure 7:**
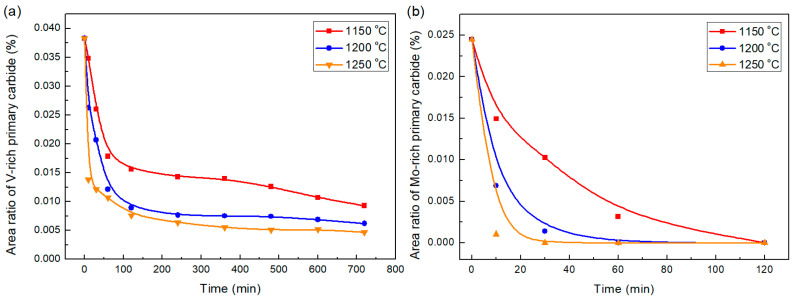
Variation in area ratios of primary carbides with soaking time at different temperatures: (**a**) V-rich primary carbides and (**b**) Mo-rich primary carbides.

Figure 7 depicts the area ratio (100 × A_PC_/A_M_, A_PC_ is the total area of primary carbide in the statistical field, A_M_ is the total statistical field area) evolution with the soaking time at different temperatures. It can be seen that the area ratios of V-rich and Mo-rich primary carbides drop with the soaking time, indicating that the primary carbides in H13 steel will dissolve in the investigated temperature range. According to the results reported by Mao et al. [28], the partial liquid phase will be formed in the inter-dendritic regions around the primary carbides above 1150 °C due to the elemental segregation. The liquid phase region increases with holding temperature, with the most pronounced increment in the holding temperature range from 1150 to 1200 °C. As the diffusion of alloying elements in the liquid phase is faster than that in the solid phase, the partial liquid phase will also provide the dissolution of primary carbide with better kinetic conditions.

**Figure 8:**
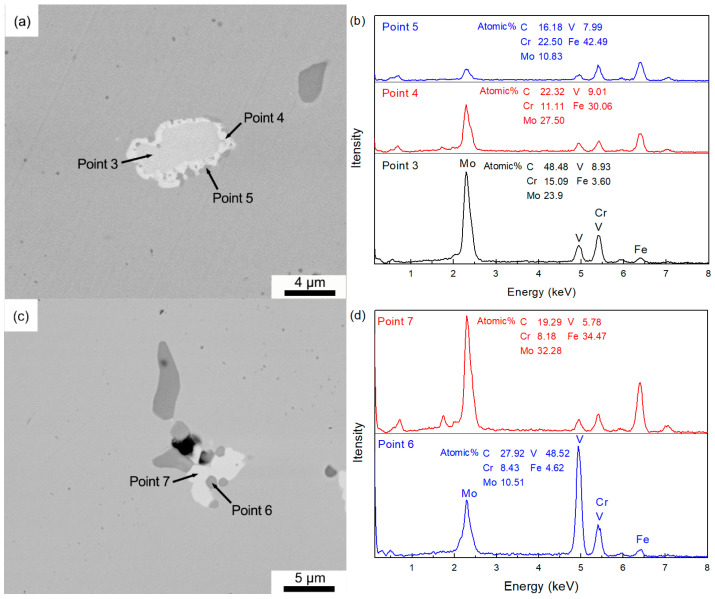
Morphology and composition evolution of Mo-rich primary carbides: (**a**,**b**) SEM images and EDS scanning of carbide soaked at 1150 °C for 10 min; (**c**,**d**) SEM images and EDS scanning of carbide soaked at 1200 °C for 10 min.

The V-rich primary carbides dissolve quickly in the first 60 min, and this process slows down, as shown in Figure 7a. In the investigated homogenization temperature and time range, the V-rich primary carbides cannot dissolve completely. As to the Mo-rich primary carbides, they could be effectively eliminated at the investigated homogenization temperature range. The main reason is that the Mo-rich M_2_C-type primary carbide is metastable phase [29,30,31]. It will start to decompose around 1000 °C, and completely transform into MC and M_6_C above 1100 °C [29]. As shown in Figure 8, when soaked at 1150 °C for 10 min, the Mo-rich primary carbides evolve into composite phases exhibiting three distinct contrasts. As the EDS scanning results (Figure 8b) show, the outer bright phase (point 4) has higher Fe and less C content which should be the M_6_C that is evident to nucleate on the interface of M_2_C/γ-Fe [31]. The gray phase is the MC that is usually formed in the interface of M_6_C/γ-Fe or M_2_C/M_6_C [31]. The decomposition of M_2_C relies on the diffusion of alloying elements. The increasing soaking temperature accelerates the decomposition. The Mo-rich primary carbide soaked at 1200 °C for 10 min, as shown in Figure 8c,d, already decomposes completely. M_6_C in H13 steel normally dissolves above 870 °C due to its narrow thermodynamic stability region. Elemental segregation can increase its stability [32]. However, the elemental segregation degree reduces with increasing soaking time. Thus, the Mo-Fe-rich M_6_C finally dissolves completely during homogenization.

**Figure 9:**
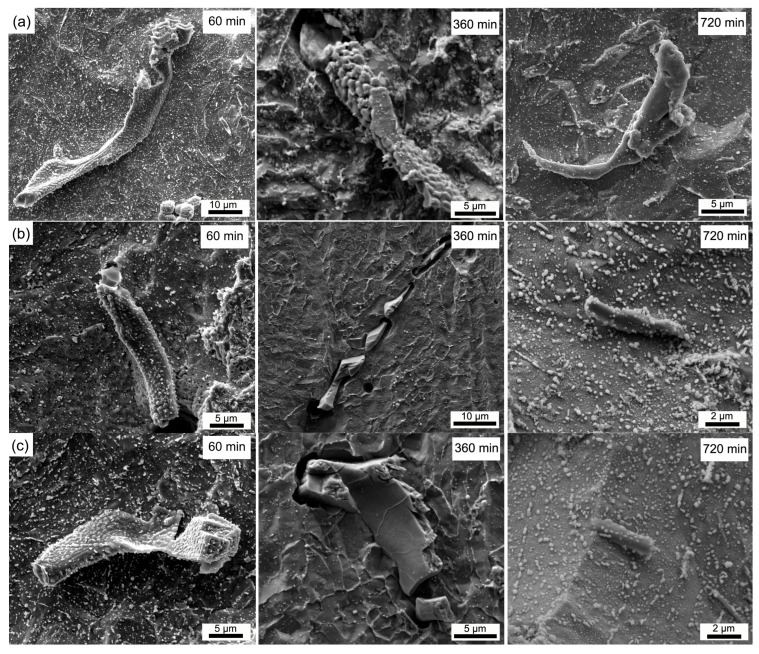
Evolution of the 3D morphology of V-rich primary carbide in the H13 steel during homogenization process at different temperatures: (**a**) 1150 °C, (**b**) 1200 °C, and (**c**) 1250 °C.

Figure 9 shows the 3D morphology evolution of V-rich primary carbides in samples soaked at 1150, 1200, and 1250 °C for 60, 360, and 720 min, respectively. The morphology evolution of primary carbides during homogenization undergoes the processes of shrinking, passivation, fragmentation and dissolution [20]. The surfaces of carbides soaked for 60 min are quite rough. When soaked at 1150 °C for 360 min, the surfaces of primary carbides exhibit a cellular morphology. Nevertheless, the carbides become smooth when soaked at 1200 and 1250 °C for 360 min. With the soaking time increased to 720 min, the surfaces of all V-rich primary carbides in samples soaked at 1150, 1200, and 1250 °C become smooth. The rough and cellular morphology may be because of the selective dissolution or electrochemical corrosion due to the composition difference in primary carbides. With the increasing soaking time, the composition of primary carbides becomes homogeneous and the surface becomes smooth gradually. At the same time, the long-strip shape primary carbides become short-stick ones after being soaked at 1200 and 1250 °C for 720 min. The long-strip primary carbides still exist after being soaked at 1150 °C for 720 min.

**Figure 10:**
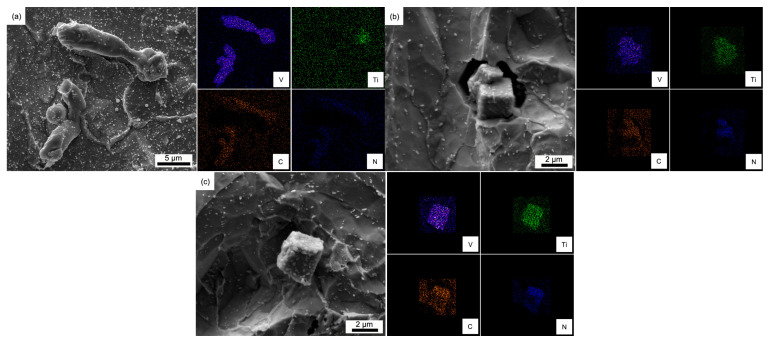
SEM images and elemental mapping of cubic primary carbides soaked for 720 min at (**a**) 1150 °C, (**b**) 1200 °C, and (**c**) 1250 °C.

It is worth noting that numerous cubic-shaped carbides are observed among the undissolved V-rich primary carbides. In the sample soaked at 1150 °C for 720 min, a stick-like primary carbide nucleating on the cubic-shaped primary carbide bearing Ti and N is found, as shown in Figure 10a. Furthermore, as the soaking temperature increases to 1200 and 1250 °C, single cubic-shaped primary carbides are exposed, as shown in Figure 10b,c, which are identified as (V, Ti) (C, N). Table 1 shows the variations in average maximum size (Amax-size), average minimum size (Amin-size), and ratio of average maximum size to average minimum size (max/min-ratio) in H13 steel during homogenization. It can be seen that both the Amax-size and Amin-size decrease during the homogenization process due to the dissolution of carbides and diffusion of segregated alloying elements. Increasing soaking temperature helps to refine the primary carbides. The max/min-ratio can demonstrate the shape of the primary carbide. It also decreases with increasing soaking time and temperature, which indicates that the long-strip primary carbides have a tendency to spheroidize during the homogenization process. At the same time, the cubic (V, Ti) (C, N) precipitate with high thermal stability has a lower max/min-ratio as well, which also contributes to the decreasing max/min-ratio.

**Figure 11:**
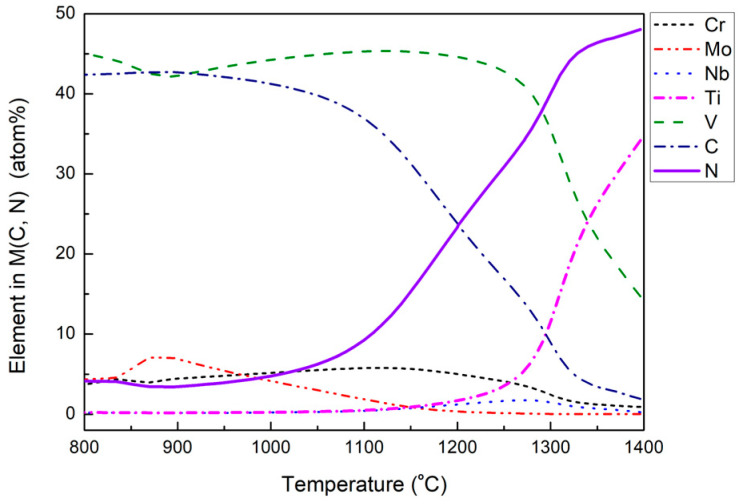
Variation in atomic percent of elements in M (C, N) phase in H13 steel with temperature.

To clarify why (V, Ti) (C, N) precipitates appear in the later stage of the homogenization process, the composition evolutions of the M (C, N) phase of the investigated H13 steel were simulated by the JMatPro 7.0 software, as shown in Figure 11. It needs to be noticed that the calculation results were based on the equilibrium conditions, which are different from the non-equilibrium conditions during the solidification process. However, it still helps to understand the evolutions of primary carbides with the soaking temperature changes. It can be seen from Figure 11 that Ti and N contents in M (C, N) phase increase with increasing soaking temperature. It implies that V-rich primary carbides with high Ti and N contents are the first to precipitate during the solidification of H13 steel. Moreover, the V-rich primary carbides containing less Ti and N contents will precipitate later. The later formed V-rich primary carbides can precipitate on the pre-formed ones. In other words, the cubic primary carbides could act as the nucleation core for the strip-like ones, as shown in Figure 10a. The later formed primary carbide would dissolve during the homogenization process if the soaking temperature exceeds the stable temperature, liberating and revealing the pre-formed primary carbide. As soaking temperature increases to 1200 and 1250 °C, most of the V-rich primary carbides dissolve in the matrix of steel, which reveals the pre-formed (V, Ti) (C, N), as shown in Figure 10b,c.

**Figure 12:**
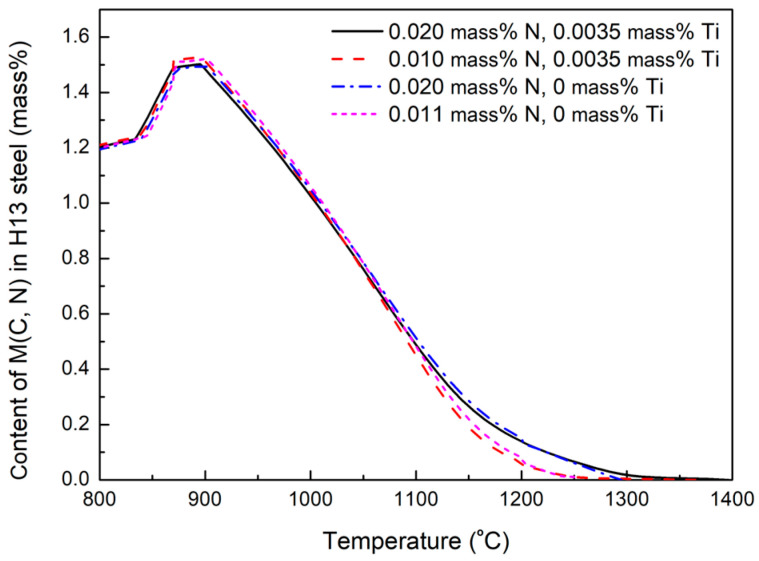
Dissolution temperature of M (C, N) phase in H13 steel with different N and Ti content.

Figure 12 shows the equilibrium content evolution of the M (C, N) phase in the H13 steel calculated by the JMatPro 7.0 software. The temperature required for the complete dissolution of V-rich primary carbides containing 0.020 mass% N and 0.0035 mass% Ti is 1396.3 °C according to the calculated results in Figure 12. Ti and N contents strongly influence the complete dissolution temperature of V-rich primary carbides. As shown in Figure 12, the decrease in Ti and N content in the H13 steel would reduce the dissolution temperature of primary carbide. The V-rich primary carbides would dissolve completely at 1250 °C when the steel contained 0.011 mass% N and no Ti. Not only are the (V, Ti) (C, N) precipitates hard to be eliminated, but they also can act as nucleation cores of long-strip V-rich primary carbides during the solidification process and promote the growth of V-rich primary carbides. Thus, in the production process, there should be a comprehensive consideration of the Ti and N microalloying effects on the mechanical strength of H13 steel and the precipitation of primary carbides in the H13 steel.

### 3.3. Grain Growth Behavior of H13 Steel During Homogenization Process

**Figure 13:**
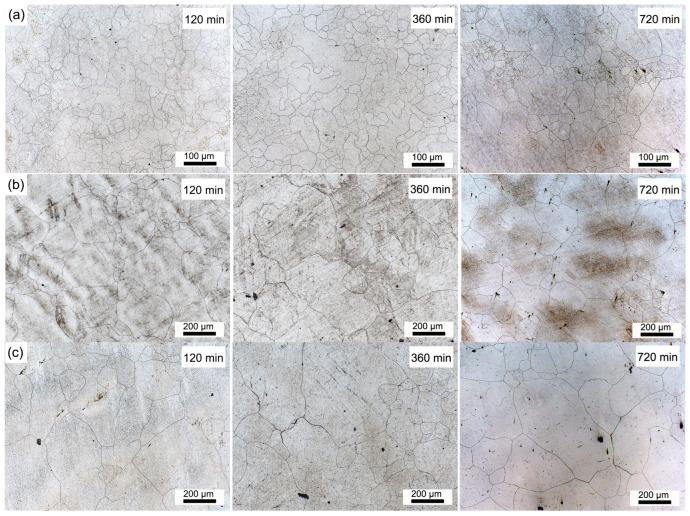
Austenite grain boundaries of H13 steel soaked at different temperatures: (**a**) 1150 °C, (**b**) 1200 °C, and (**c**) 1250 °C.

**Figure 14:**
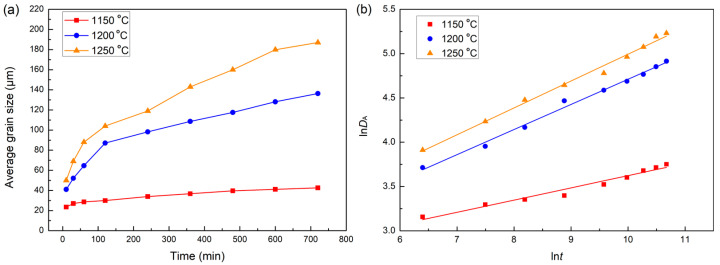
(**a**) Relationship between the average grain size of austenite and soaking time and (**b**) plots of ln*D_A_* versus ln*t*.

Figure 13 shows the austenite grain boundaries of samples heated at 1150, 1200, and 1250 °C for 120, 360, and 720 min. The austenite grains of samples become coarser with the soaking time. At the same time, the austenite grains of samples heated at 1200 and 1250 °C are significantly larger than those heated at 1150 °C. The relationship between the average grain size and the soaking time at different temperatures is depicted in Figure 14a. The grains grow slowly at 1150 °C, which is in concert with previous findings [33], whereas they grow fast when the soaking temperature is increased to 1200 °C. In addition, the austenite grains grow quickly in the first, and then the growth rate slows down gradually with the increasing soaking time. The driving force of the grain growth is believed to be the decreasing surface energy of the grains. However, the driving force will decrease with the migration of grain boundaries [34]. Consequently, the grain growth rate decreases with increasing soaking time. The grain growth kinetics of steel can be described with Beck’s classical theory as follows [33,34,35,36]:

(8) $$D_{A}=k\cdot t^{n}$$

where *D*_A_ is the average grain size of austenite grain (μm), *t* is the soaking time (s), *k* is the grain growth rate constant, and *n* is the grain growth exponent. According to Equation (8), ln*D*_A_ and ln*t* can be linearly fitted, as shown in Figure 14b. The slopes of 1150 to 1250 °C are 0.138, 0.283, and 0.303, respectively. The relationship between austenite grain growth and the austenitizing condition is explained by the thermally activated atomic jump process. Thus, Equation (8) can be further expressed by Equation (9) as follows [37,38]:

(9) $$D_{A}=k_{1}\cdot t^{n}\cdot \exp\left(-\frac{Q_{gg}}{RT}\right)$$

where *k*_1_ is constant, and *Q*_gg_ is the activation energy for grain boundary migration (J·mol^−1^). Considering the great difference between grain size soaked at 1150 and that above 1200 °C, the average grain size is fitted in section by temperature according to Equation (9).

The austenite grain growth model in the soaking temperature range of 1150–1200 °C and 1200–1250 °C can be obtained by multiple regressions and expressed as Equations (10) and (11), respectively. The activation energy for grain boundary migration significantly reduces when soaking temperature is higher than 1200 °C. The precipitates dissolve quickly when soaked above 1200 °C which suppresses the pinning effect of precipitates on grain boundaries. In addition, the diffusion of alloying elements is enhanced which suppresses the solute drag effect. These factors lead to the decrease in activation energy [38]. This results in the abnormal growth of grains above 1200 °C.

(10) $$D_{A}=8.7\times 10^{12}\cdot t^{0.211}\cdot \exp\left(-\frac{3.34\times 10^{5}}{RT}\right)\;$$

(11) $$D_{A}=1.88\times 10^{4}\cdot t^{0.290}\cdot \exp\left(-\frac{9.83\times 10^{4}}{RT}\right)$$

The experimental results indicate that, despite a slow austenite grain growth at 1150 °C, the elimination of primary carbides and dendritic structures is not quite effective. Most of the primary carbides dissolve after being soaked for 60 min at 1200 and 1250 °C. The time required for eliminating dendritic structures of the investigated H13 steel at 1250 °C is 600 min, while that at 1200 °C is higher by 1.78 times than that at 1250 °C according to the calculated results. Therefore, 1250 °C is the optimal soaking temperature for the high-temperature homogenization treatment of the investigated H13 steel. In addition, 60 min would be recommended as the minimum soaking time if the main purpose is eliminating the primary carbides. The optimal soaking time range for the complete elimination of dendritic structures in the investigated H13 steel is 600 min. Noteworthy is a rapid growth of austenite grains in H13 steel at 1250 °C. Proper forging (rolling) and subsequent heat treatment are needed for mitigating coarse grains.

## 4. Conclusions

(1) The carbide-formation alloying elements Cr, Mo, and V are significantly enriched in the inter-dendritic regions of H13 steel. The boundaries between the dendrite arms and inter-dendritic regions become blurred during the homogenization process, and disappear when soaked at 1250 °C for 600 min due to the diffusion of segregated alloying elements. Based on the established homogenization kinetics model, the soaking times for eliminating the dendritic structures at 1150 and 1200 °C are estimated to be 3.31 and 1.78 times longer than that at 1250 °C. Increasing the soaking temperature and decreasing the dendritic arms spacing can effectively shorten the soaking time.

(2) There are V-rich and Mo-rich primary carbides in H13 steel. The Mo-rich primary carbides will decompose and dissolve completely in the investigated temperature range of 1150–1250 °C. The V-rich primary carbides will partly dissolve and maintain long-strip morphology at 1150 °C due to their higher thermal stability. However, they become short-stick and Ti-N-containing-cubic carbides when soaked above 1200 °C. Decreasing the Ti and N content is beneficial for lowering the complete dissolution temperature of the primary carbides.

(3) The austenite grain growth rate in the H13 steel increases with the soaking temperature during the homogenization process. When soaked at 1150 °C, the austenite grains grow slowly. However, the grain grows abnormally above 1200 °C. The activation energy of grain boundary migration of the investigated H13 steel in the temperature range of 1150–1200 °C is 3.34 × 10^5^ J·mol^−1^, and that in the temperature range of 1200–1250 °C is 9.83 × 10^4^ J·mol^−1^.

## Figures and Tables

**Table 1:** Variations in average maximum size, average minimum size, and max/min-ratio of primary carbides during homogenization process.

Average Size	Soaked at 1150 °C			Soaked at 1200 °C			Soaked at 1250 °C		
	60 min	360 min	720 min	60 min	360 min	720 min	60 min	360 min	720 min
Amax-size (μm)	9.93	7.06	6.55	6.11	4.97	4.38	5.88	4.72	3.69
Amin-size (μm)	4.33	4.09	3.86	3.86	3.23	3.03	3.75	3.16	2.88
Max/Min-ratio	2.29	1.73	1.70	1.58	1.54	1.45	1.57	1.49	1.28

## Data Availability

The original contributions presented in this study are included in the article. Further inquiries can be directed to the corresponding authors.
